# Polychlorinated Biphenyls-Induced Oxidative Stress on Rat Hippocampus: A Neuroprotective Role of Quercetin

**DOI:** 10.1100/2012/980314

**Published:** 2012-01-04

**Authors:** Kandaswamy Selvakumar, Senthamilselvan Bavithra, Gunasekaran Krishnamoorthy, Prabhu Venkataraman, Jagadeesan Arunakaran

**Affiliations:** ^1^Department of Endocrinology, Dr. ALM Post Graduate Institute of Basic Medical Sciences, University of Madras, Chennai 600113, India; ^2^Department of Biochemistry, Asan Memorial Dental College & Hospital, Asan Nagar 603105, India; ^3^Department of Medical Research, SRM Medical College & Hospital, SRM University, Kattankulathur 603203, India

## Abstract

Present study is aimed to evaluate the ameliorative role of quercetin on PCBs-induced oxidative stress in hippocampus of Wistar rats. *Group I* rats received vehicle (corn oil) intraperitoneally (i.p); *Group II* received quercetin 50 mg/kg bwt/day (gavage); *Group III* received PCB 2 mg/kg bwt/day (i.p); *Group IV* received PCB (i.p) and simultaneously quercetin through gavage. After 30 days, rats were euthanized and hippocampus was dissected from each rat brain. Oxidative stress was assessed by determining the levels of H_2_O_2_, LPO, Pcc, and alteration in the functional markers such as CK, AchE, and ATPases activities in the hippocampus of control and experimental animals. A significant increase in the levels of stress markers and decrease in level of functional markers were observed in PCBs-treated rats. Moreover DNA fragmentation and histological studies were ascertained to confirm PCBs toxicity. In conclusion, quercetin shows a protective role against PCBs-induced oxidative damage in rat hippocampus.

## 1. Introduction

Polychlorinated biphenyls (PCBs) are members of halogenated aromatic environmental pollutants that have been identified in diverse environmental matrices [1]. About 50% of technical PCBs mixtures produced worldwide were utilized as capacitor and transformer insulating oils, while the remaining part has found diverse use in hundreds of applications and high residue levels are still detected in human tissues [2]. PCBs are distributed throughout the entire ecosystem including soil, air, and water. They are also likely to bioaccumulate in the food chain because of their lipophilicity and therefore belong to a class of environmental chemicals called persistent bioaccumulative toxicants (PBTs) [3]. PCBs induce subtle and long-lasting neurological damages resulting in the impairment of motor and learning abilities [4]. Problems associated with PCB include hypothyroxinemia, spatial learning and memory deficits, neurochemical and neurobehavioral alterations, and reproductive alterations [5].

Epidemiological and experimental data confirm that exposure to PCBs causes negative impact on neuropsychological functions causing cognitive and psychomotor deficits in children and experimental animals [6]. PCBs can induce neurotoxic effects including morphological changes of neurons and developmental defects of the nervous system, which may lead to neurobehavioral and intellectual disorders as well as induce brain tumor promoting factors [7]. These functional deficits reflect subtle perturbations of neuronal connectivity in the developing brain; however the cellular and molecular mechanisms by which PCBs interfere with neuronal connectivity remain an area of active investigation [8]. PCBs have been shown to increase Reactive Oxygen Species (ROS) in primary neuronal cell cultures [9] which may be linked to increased neuronal apoptosis. The hippocampus has long been implicated in memory functions for human and other mammals are much vulnerable to oxidative damage, due to its high oxygen consumption, higher levels of polyunsaturated fatty acids (PUFAs) which may lead to various neurodegenerative diseases. Prenatal exposure to PCB95 (2,2′,3,5′6-pentachlorobiphenyl) and the commercial mixture Aroclor 1254, in which noncoplanar congeners are more abundant, were shown to persistently alter excitability and long-term potentiation in rat hippocampal slices *in vitro *[10].

Oxidative modification of brain proteins may disturb neuronal functions by decreasing the activities of key metabolic enzymes and affecting cellular signaling systems [11]. Protein oxidation is induced either directly by ROS or indirectly by reaction with secondary by-products of oxidative stress [12]. The most common products of protein oxidation in biological samples are the protein carbonyl derivatives of Pro, Arg, Lys, and Thr. These derivatives are chemically stable and serve as markers of oxidative stress for most types of ROS-incited protein oxidation [13]. PCBs induce oxidative stress in rat brain by decreasing the activities of antioxidant enzymes, altering membrane bound ATPases and cholinergic function [14]. The cholinergic system plays a crucial role in cognitive function in which choline esterases are ubiquitous constituents. Acetylcholine (Ach), an important neurotransmitter, depends on the activity of acetylcholinesterase (AchE) which is involved in the release of Ach [15]. Tsakiris [16] showed that the activity of AchE was inhibited by free radical formation. This may upset the pro-oxidant/antioxidant balance within the brain, which could be one of the reasons for decreased AchE activity. Creatine kinase (CK) which plays a key role in energy metabolism of nervous tissue is sensitive to oxidative damage and might be one of the targets for ROS in the brain of neurodegenerative diseases [17].

 Quercetin is a flavonoid, which is present in fruits, vegetables, and several other dietary sources [18]. It is marketed as a diet supplement with antihistamine, anti-inflammatory, antiviral, immunomodulatory, and antioxidant properties [19]. It also possesses antifungal, vasorelaxation activities on hippocampal neurons [20]. It scavenges superoxide in ischemia-reperfusion injury. Previous report suggested that ROS scavenging activity of quercetin on PCBs exposure might be due to superoxide anion and hydroxyl radical alteration by quercetin [21]. Quercetin can interfere with the production of ROS and acts through two mechanisms to reduce tissue damage by chelating effect and by nullifying the lipid peroxidation (LPO). With this background, the present study was designed to determine the impact of quercetin on PCBs-induced alterations in oxidative stress markers, DNA fragmentation, and the activities of functional markers in hippocampus of adult rats.

## 2. Materials and Methods

### 2.1. Chemicals

Aroclor 1254 was purchased from Chem Services, West Chester, PA (USA). Quercetin and all other molecular grade chemicals were purchased from Sigma-Aldrich Pvt. Limited (USA).

### 2.2. Animals

Healthy adult male albino rats of Wistar strain *Rattus norvegicus *weighing about 180–200 g (90 days) were used in the present study. The study protocol was reviewed and approved by the institutional ethical committee (Ref No. IAEC No: 03/014/09). The animals were housed in clean polypropylene cages, maintained in air-conditioned animal house with constant photoperiod of 12 h light/dark cycle. The animals were fed with pellet diet (Gold Mohur Ltd., Mumbai, India) and drinking water *ad libitum.*

### 2.3. Treatment Procedure

Rats were assigned into four groups, six animals in each group, and the treatment was given regularly at 10.30 am daily for 30 days. Body weights of the animals were monitored throughout the experimental period. in *Group* 1: rats were injected with corn oil (80 *μ*L) intraperitoneally (i.p) and maintained as control in *Group* 2: rats received quercetin by gavage at a dose of 50 mg/kg bwt [21] in *Group* 3: rats received i.p injection of Aroclor 1254 at a dose of 2 mg/kg bwt [14] in *Group* 4: rats were administered with Aroclor 1254 at a dose of 2 mg/kg bwt (i.p.) and simultaneously quercetin was supplemented by gavage at a dose of 50 mg/kg bwt. The doses of PCBs [14] Aroclor 1254 and Quercetin [21] were selected as per the literature.

### 2.4. Sample Collection and Preparation

24 h after the last treatment, the animals were sacrificed by cervical decapitation. The brain was excised immediately and washed in ice-cold physiological saline repeatedly; hippocampus was separated and weighed accurately. 10% tissue homogenate was prepared by homogenizing tissue with Tris-HCl buffer (0.1 M, pH 7.4), followed by centrifugation at 12,000 rpm for 10 min. The supernatant was used for biochemical analyses as described here in after. Protein concentration of the tissue homogenate was determined by the standard method of Lowry et al. [22] using bovine serum albumin as a standard.

### 2.5. Hydrogen Peroxide Assay

The hydrogen peroxide (H_2_O_2_) generation was assayed by the method of Pick and Keisari [23]. Horseradish peroxidase converts hydrogen peroxide into water and oxygen. This causes oxidation of phenol red forms adduct with dextrose which has maximum absorbance at 610 nm and can be recorded spectrophotometrically. Levels of H_2_O_2_ generation were expressed as mM of H_2_O_2_-generated/mg protein.

### 2.6. Lipid Peroxidation Assay

Tissue lipid peroxidation (LPO) was measured by the method of Devasagayam and Tarachand [24]. Malondialdehyde, an end product of lipid peroxidation reacts with thiobarbituric acid (TBA) to form a pink chromogen (TBA 2-malondialdehyde adduct) and was measured by its absorbance at 532 nm with a spectrophotometer. The results were expressed as nmoles of malondialdehyde (MDA)/mg protein.

### 2.7. Protein Carbonyl Group Estimation

The protein carbonyl content was determined by the method of Levine et al. [25]. The hippocampus tissue homogenate was divided into two portions containing 1 mg protein each. To one portion, equal volume of 2 N HCl was added and incubated at room temperature for one hour, with intermittent shaking. The other portion was treated with an equal volume of 10 mM DNPH in 2 N HCl and incubated for one hour at room temperature. After incubation, the mixture was precipitated with 10% TCA and centrifuged. The precipitate was washed with ethanol: ethylacetate (1 : 1) twice, dissolved in 1 mL of 6 M guanidine HCl, centrifuged at low speed, and the supernatant was taken. The difference in absorbance between the DNPH-treated and HCl-treated samples was determined at 366 nm and the results were expressed as nmoles of carbonyl groups/mg of protein. BSA (1 mg/mL) was used as a standard.

### 2.8. Acetyl Cholinesterase Activity

The acetyl cholinesterase (AchE) activity was measured by providing an artificial substrate acetylthiocholine iodide (ATCI) according to Ellman et al. [26]. Thiocholine, the cleavage product of ATCI, was allowed to react with the-SH reagent 5,5′-dithiobis-(2-nitrobenzoic acid) (DTNB), which is reduced to thionitrobenzoic acid, a yellow-colored anion with an absorption maximum at 412 nm. The extinction coefficient of the thionitro benzoic acid is 1.36 × 104/molar/cm. The concentration of thionitrobenzoic acid detected using a UV spectrophotometer was then taken as a direct estimate of the AChE activity. The enzyme activity was expressed as moles of substrate hydrolyzed/min/mg protein.

### 2.9. Creatine Kinase Activity

Creatine Kinase (CK) activity was estimated by the method of Okinaka et al. [27]. Creatine kinase catalyses the conversion of creatine to creatine phosphate. Phosphate reacts with ammonium molybdate to form phosphomolybdate. The hexavalent molybdenum of phosphomolybdate is reduced by ANSA to give blue-colored complex, which is measured at 640 nm. The enzyme activity is expressed as *μ*moles of phosphorous-liberated/min/mg protein.

### 2.10. Evaluation of ATPases

The activities of Na^+^/K^+^ ATPase [28], Ca^2+^ ATPase [29], and Mg^2+^ ATPase [30] were measured by evaluating the inorganic phosphorous (Pi) liberated by splitting of ATP molecules by ATPase in the presence of Na^+^/K^+^ ions, Ca^2+^ions, and Mg^2+^ ions, respectively. Phosphate reacts with ammonium molybdate to form phosphomolybdate. The hexavalent molybdenum of phosphomolybdate is reduced by ANSA to give blue colour complex, which is measured at 620 nm. Enzyme-specific activity was expressed as nmoles Pi-released/min/mg of protein.

### 2.11. DNA Fragmentation

100 mg hippocampus tissue was homogenized in 1 mL 1X suspension buffer in 2 mL microcentrifuge tube. After homogenization, 5 *μ*L RNase solution (10 mg/mL) was added and mixed 5-6 times by inverting the vial. Then the content was incubated at 65°C for 10 min with intermittent mixing. After incubation, 1 mL lysis buffer was added, mixed, and incubated again at 65°C for 15 min and then the lysate was cooled at RT. Lysate was centrifuged at 13,000 rpm for 1 min at RT. To the supernatant, equal volume of isopropanol was added, mixed well, and centrifuged at 13,000 ×g for 15 min at RT. To the pellet, 0.5–1 mL of 70% ethanol was added and centrifuged at 13,000 ×g for 15 min at RT, the pellet was removed, and the step was repeated thrice. The pellet was then dried at 37°C for 10 min. 50 *μ*L of autoclaved milliQ water was added and the DNAs was suspended by placing the vial at 4°C overnight. The isolated DNA were resolved by electrophoresis through a 1% agarose gel and stained with ethidium bromide. The resolved fragments of DNA in the agarose gel were scanned with a Gel Doc image scanner (Bio-Rad, USA).

### 2.12. Histology

The same groups were maintained for histological study. Animals were sacrificed by perfusion. 10% formaldehyde was used for fixation. The hippocampus was separated from the whole brain, it was cleaned, and tissue was sliced into 0.5 cubic cm. After further fixation by immersion in 4% formaldehyde in PBS (pH 7.4) overnight at RT (8–12 h), washing in PBS and immersion in 70% ethanol, the tissue was maintained at 37°C until the embedding in paraffin was made. The paraffin blocks were cut into 10 *μ*m thickness using rotary microtome. The sections were stained with haematoxylin and eosin [31]. The hippocampus morphology was analyzed by Nikon Microscope Eclipse 80i (10x and 40x magnifications).

### 2.13. Statistical Analysis

All values were expressed as mean ± SEM of six animals. Data were analyzed using one-way analysis of variance (ANOVA) followed by post hoc test Student's Newman-Keul's test (SNK) with Graphpad Prism5 software. In all cases, *P* < 0.05 was considered as statistically significant.

## 3. Results

### 3.1. Body Weight and Relative Hippocampal Weight of PCBs-Exposed Adult Rats

**Figure 1:**
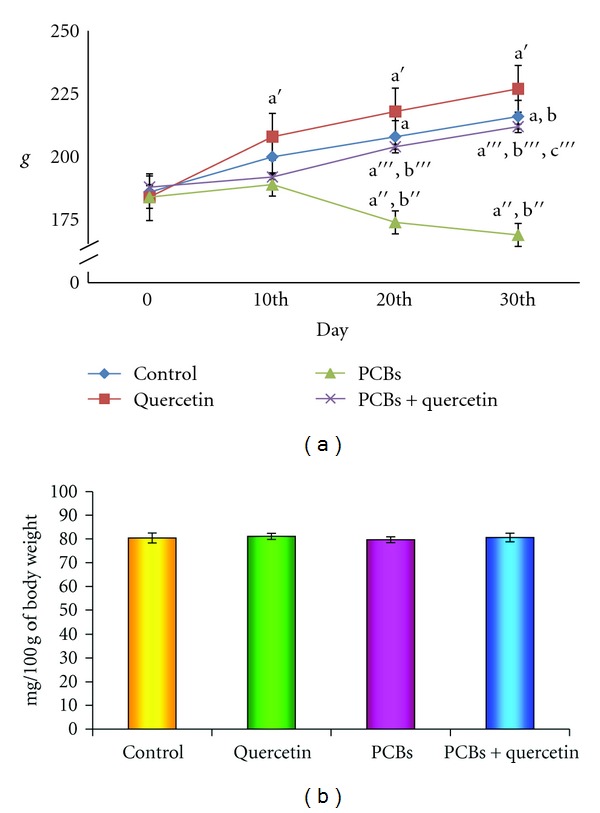
(a), (b): Effect of quercetin on body weight and relative hippocampal weight of PCBs-exposed adult rats. Each bar represents mean ± SEM of 6 animals. Statistical significance is at *P* < 0.05 (a: Control versus others; b: Quercetin versus PCB, PCB + Quer; c: PCB versus PCB + Quer). a: Control 0 Day versus 10th day, 20th day, and 30th day; b: Control 10th day versus 20th day and 30th day; a': Quercetin 0 day versus 10th day, 20th day, and 30th day; b':Quercetin10th day versus 20th day and 30th day; a”: PCB 0 day versus 10th day, 20th day, and 30th day; b”: PCB 10th day versus 20th day and 30th day; a”': PCB + Quercetin 0 day versus 10th day, 20th day, and 30th day; b”': PCB + Quercetin10th day versus 20th day and 30th day; c”': PCB+Quercetin 20th day versus 30th day.

The effect of PCBs on change in body weight of rats is shown in Figures 1(a) and 1(b). No mortality was observed in any of the experimental groups. Maximum weight gain was observed in the quercetin-administered rats as compared to all other groups. There was a gradual and significant (*P* < 0.05) decrease in body weight of PCBs intoxicated rats as compared to control and quercetin-treated rats. No significant change was observed in the relative hippocampal weight of control and treated groups.

### 3.2. Hydrogen Peroxide Generation

**Figure 2:**
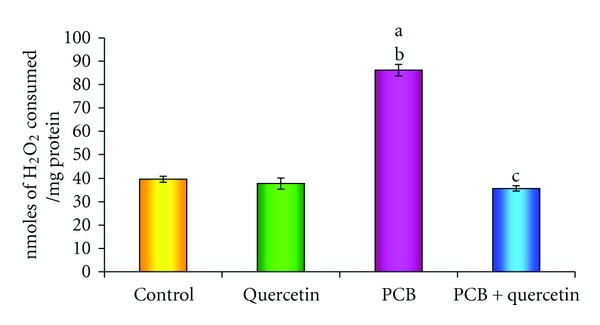
Effect of quercetin on H_2_O_2_ generation in hippocampus of PCBs-exposed adult rats. Each bar represents mean ± SEM of 6 animals. Statistical significance is at *P* < 0.05 (a: Control versus others; b: Quercetin versus PCB, PCB + Quer; c: PCB versus PCB + Quer).

Figure 2 shows the PCBs intoxication-induced generation of hydrogen peroxide in hippocampus. The level of hydrogen peroxide, a readily diffusible free radical, is found to be increased significantly in the hippocampus of PCBs-exposed rats whereas the simultaneous administration of quercetin as well, quercetin alone treated group decreased the level.

### 3.3. Effect on Lipid Peroxidation

**Figure 3:**
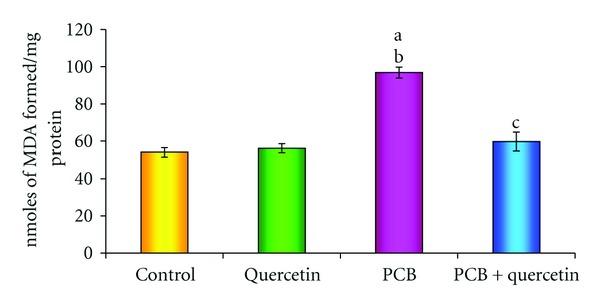
Effect of quercetin on lipid peroxidation in hippocampus of PCBs-exposed adult rats. Each bar represents mean ± SEM of 6 animals. Statistical significance is at *P* < 0.05 (a: Control versus others; b: Quercetin versus PCB, PCB + Quer; c: PCB versus PCB + Quer).

In order to verify the presence of oxidative imbalance induced by PCBs, levels of lipid peroxidation were measured in all groups (Figure 3). The increased formation of MDA measured in PCBs intoxicated rats compared with the control group is an index of LPO (*P* < 0.05). No significant change was observed in the LPO level of control and quercetin-treated rats. Daily treatments with quercetin significantly lessen the PCBs-induced lipid peroxidation in rat hippocampus.

### 3.4. Effect on Protein Carbonyl Formation

**Figure 4:**
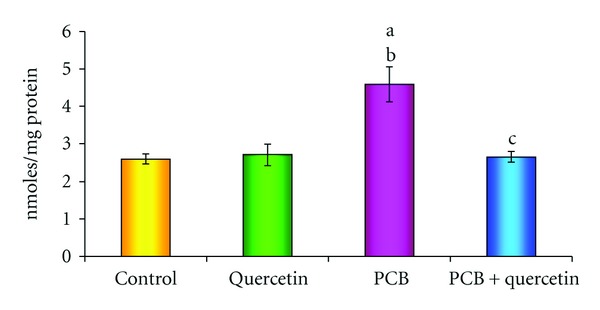
Effect of quercetin on protein carbonyl content in hippocampus of PCBs-exposed adult rats. Each bar represents mean ± SEM of 6 animals. Statistical significance is at *P* < 0.05 (a: Control versus others; b: Quercetin versus PCB, PCB + Quer; c: PCB versus PCB + Quer).

Figure 4 depicts the effect of PCBs intoxication on protein carbonyl formation in hippocampus. Protein carbonyl, chemically stable oxidative stress marker formation was significantly increased in PCBs-treated rats as compared to control rats. However, coadministration of quercetin along with PCBs showed inhibition of protein carbonyl formation comparable to PCBs treated rats.

### 3.5. Activities of Creatine Kinase

**Figure 5:**
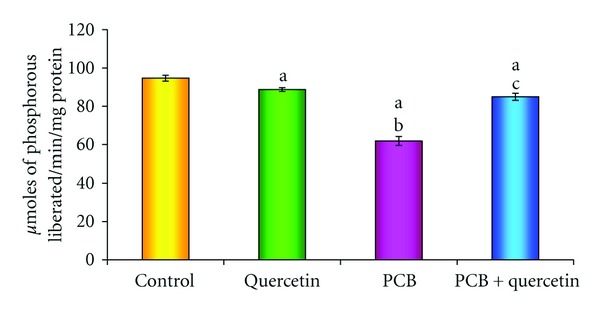
Effect of quercetin on creatine kinase in hippocampus of PCBs-exposed adult rats. Each bar represents mean ± SEM of 6 animals. Statistical significance is at *P* < 0.05 (a: Control versus others; b: Quercetin versus PCB, PCB + Quer; c: PCB versus PCB + Quer).

Figure 5 proclaims the effect of quercetin on creatine kinase (CK) activity in hippocampus of PCBs-exposed adult rats. The CK activity which plays a key role in energy metabolism of nervous tissue is significantly decreased in PCBs treated rats. However, simultaneous supplementation of quercetin restored the same. Quercetin alone treatment did not show any change.

### 3.6. Effect on AchE Activity

**Figure 6:**
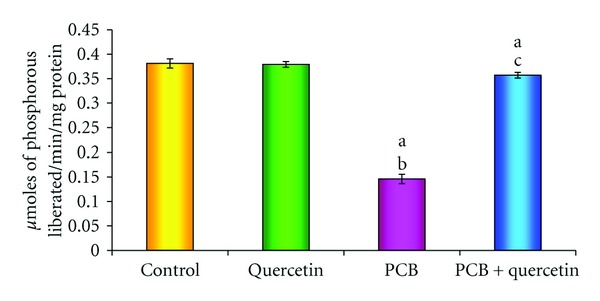
Effect of quercetin on acetyl cholinesterase in hippocampus of PCBs-exposed adult rats. Each bar represents mean ± SEM of 6 animals. Statistical significance is at *P* < 0.05 (a: Control versus others; b: Quercetin versus PCB, PCB + Quer; c: PCB versus PCB + Quer).

Significant reduction was observed in activity of acetylcholine esterase of PCBs-treated group, whereas quercetin given simultaneously with PCBs retrieved the enzyme activity as that of control. Quercetin alone treatment did not show any significant change (Figure 6).

### 3.7. Effect on Na^+^/K^+^, Mg^2+^, and Ca^2+^-ATPases

**Figure 7:**
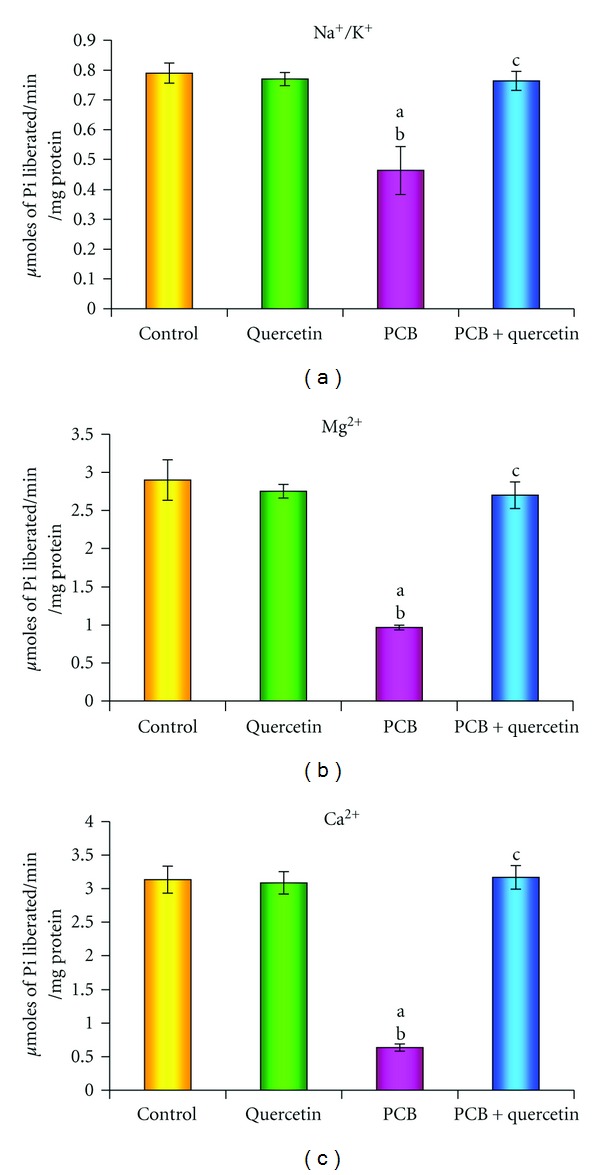
(a), (b), (c): Effect of quercetin on Na^+^/K^+^, Mg^2+^, and Ca^2+^-ATPases activities in hippocampus of PCBs-exposed adult rats. Each bar represents mean ± SEM of 6 animals. Statistical significance is at *P* < 0.05 (a: Control versus others; b: Quercetin versus PCB, PCB + Quer; c: PCB versus PCB + Quer).

The effect of PCBs administration on ATPases such as Na^+^/K^+^, Mg^2+^ and Ca^2+^- ATPases, which play a vital role in active transport of ions in hippocampus is shown in Figures 7(a), 7(b), and 7(c). A significant decrease in Na^+^/K^+^, Mg^2+^ and Ca^2+^-ATPases activities was recognized in the PCBs-treated rats compared to control. Simultaneous quercetin treatment along with PCBs brought back all the activities as that of control. Quercetin alone treatment did not show any significant change.

### 3.8. DNA Fragmentation

**Figure 8:**
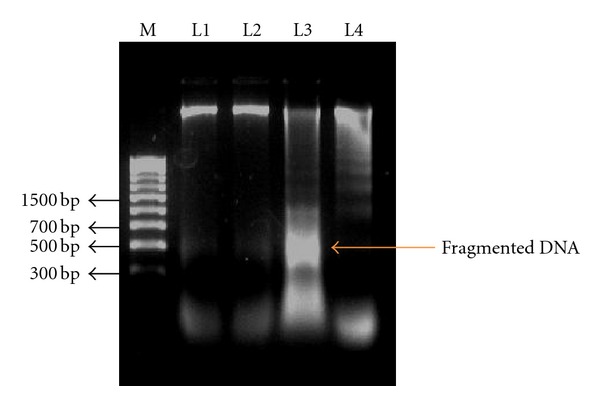
Effect of quercetin on DNA fragmentation in hippocampus of PCBs-exposed adult wister rats. Each bar represents mean ± SEM of 6 animals. Statistical significance is at *P* < 0.05 (a: Control versus others; b: Quercetin versus PCB, PCB + Quer; c: PCB versus PCB + Quer). M: 100bp Marker; L1: Control; L2: Quercetin; L3: PCB; L4:PCB+Quercetin.

To confirm the toxicity caused by oxidative stress due to PCBs exposure, fragmentation of DNA has been observed.  Figure 8 represents the agarose gel pattern of fragmented DNA of hippocampus of PCB treated rats. The fragmentation of DNA showed a great increase in the PCBs-exposed hippocampus but restored in the simultaneous supplementation of quercetin-treated hippocampus. Quercetin alone treated group did not show any change.

### 3.9. Histological Changes in Hippocampus

**Figure 9:**
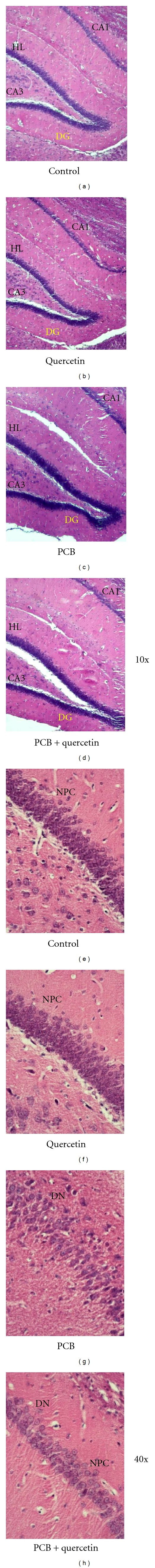
Photomicrograph of the hippocampal pyramidal cell layers in Control, Quercetin, PCB, and PCB with Quercetin Treated rats. Histology of hippocampus in PCBs-exposed adult rats. Figures (a), (b), (c), (d) are 10x magnification, (e), (f), (g), and (h) are 40x magnification. The normal pyramidal cells (NPC) of hippocampal layer are normal in control rats (a) (e) and quercetin alone treated rats (b) (f). Degeneration of pyramidal cells is observed in PCB-treated rats (c) (10x). Degenerative neurons (DNs) as well degenerative layers are also observed in PCB-treated rats (40x) (g). Restoration of pyramidal cells in both 10x and 40x magnifications (d) (h) few degenerated pyramidal cells are also seen (d) (h). (Haematoxylin and Eosin staining 10x/40x). HL- Hippocampal Layer DN- Degenerative Neurons NPC- Normal Pyramidal Cell DG- Dentate gyrus CA1, CA3- Cornus Ammonis 1, Cornus Ammonis 3.

Figures 9(a) and 9(e) depicts, the 10x and 40x magnifications of hippocampus in control rats. The pyramidal cells of hippocampal layer were normal in control animals. Figures 9(b) and 9(f) show the same magnification in quercetin alone treated rats. The normal pyramidal cellular arrangement of hippocampus was observed in both magnifications of quercetin alone treated animals. Figures 9(c) and 9(g) shows the degeneration of pyramidal cells. The degenerative neurons were observed in both magnifications, in hippocampus of PCB treated rats. Figures 9(d) and 9(h) show the pyramidal cells of hippocampal layer in simultaneous administration of quercetin with PCB-exposed rats. Restoration of pyramidal cell was observed. Few degenerated pyramidal cells were also seen.

## 4. Discussion

Several studies show that PCBs generate transient ROS [32]. PCBs, especially higher chlorinated PCBs, may selectively induce cytochrome P450s as a possible source of ROS [33] or, alternatively, the oxidation of a broad range of endogenous and exogenous substances. Some studies suggest that oxidative stress induced by PCBs is due to the interaction of these compounds with aryl hydrocarbon receptors (AhRs) and activation of the cytochrome P450 IA subfamily. The cytochrome P450 catalyzed oxidation of lower chlorinated biphenyls gives rise to mono-and di-hydroxy metabolites, which then auto-oxidize or get enzymatically oxidized to semiquinones and/or quinines. Some PCB quinones undergo redox cycling with the formation of ROS, thus becoming another source of oxidative stress. ROSs like O_2_^•−^, HO^•^, and H_2_O_2_ are thought to contribute to LPO, DNA damage, and protein degradation [34]. PCB induces oxidative stress, decreases the activities of antioxidant enzymes, and causes disruption in the functional parameters of ventral prostate, testicular Leydig and Sertoli cells [35–40]. The present study is also consistent to the same observations in the hippocampus.

 Twaroski et al. [41] indicated that toxic manifestation induced by PCB may associate with enhanced production of ROS and thereby induce oxidative stress through the initiation of self-propagating LPO reaction. A study showed that hippocampus is more vulnerable to tertbutyl hydroperoxide t-BuOOH-induced oxidative insult than mid brain. This may be due to a higher level of arachidonic acid metabolism in the hippocampus, which generates oxygen radicals as a byproduct [42]. Oxidative modification of proteins *in vivo *may affect a variety of cellular functions involving proteins: receptors, signal transduction mechanisms, transport systems, and key metabolic enzymes. It could also contribute to secondary damage to other biomolecules, for instance, inactivation of DNA repair enzymes and loss of fidelity of DNA polymerases in replicating DNA [43]. The increased protein carbonyl formation in PCBs treated group proves that ROS produced by PCB had oxidized the proteins and may impair the cellular functions. Simultaneous supplementation of quercetin had inhibited the protein carbonyl formation by scavenging the ROS.

 Nguon et al. [44] studied that activation of creatine kinase (CK) system and changes in CK expression may be earlier indicator of oxidative stress in the cell. A decrease in hippocampal CK on the exposure of PCB might be due to the leakage of this enzyme from the tissue into the blood stream. Earlier studies in our laboratory stated that PCBs induced free radicals and other ROSs that attack membrane lipids leading to increased permeability and altered fluidity of the membrane in different brain regions [45, 46]. This perturbation in the structural and functional integrity of the membrane could have resulted in the release of the enzymes into the circulation. Direct oxidation of sulfhydryl groups in cysteines located in the active site of CK may also inactivate the enzyme [47]. It may be also one of the reasons for the decreased CK activity in the hippocampus of PCB exposed rats. Therefore, oxidative stress is an important event that has been related to the pathogenesis of diseases affecting the central nervous system. This is understandable since this tissue is highly sensitive to oxidative stress due to its high oxygen consumption, its high iron and lipid contents, especially poly-saturated fatty acids, and the low activity of antioxidant defenses [48].

 Carbonell and Rama [49] showed that the activity of AchE was inhibited by free radical formation. This might upset the pro-oxidant and antioxidant balance within the brain, which could be one of the reasons for decreased AchE activity. Taskiris et al. [50] revealed that the PCB exposure affected the cholinergic system in experimental animals. Venkataraman et al. [15] also showed a decreased AchE activity after PCB exposure in brain regions such as cortex, cerebellum, and hippocampus. The present study also proved the same. Simultaneous supplementation of quercetin retrieved both CK as well as the AchE activities due to its free radical scavenging capacity.

Activities of all ATPases in hippocampus were significantly decreased in PCBs-exposed rats. Deficiency of ATP or the production of ROS inhibits the activity of Na^+^/K^+^ ATPase. Na^+^/K^+^ ATPases were decreased in experimental model of cerebral ischemia [51] and in many neurodegenerative disorders. Wyse et al. [52] studied the effects of ROS on this enzyme and it includes selective alterations of its active site. The other ATPases such as calcium and magnesium ATPases were also decreased in the hippocampus of PCBs exposed rats; hence, Ca^2+^ ATPase and Mg^2+^ are vulnerable to damage by oxy radicals. Mishra et al. [53] stated that there is alteration in calcium homeostasis of PCBs- treated rat's cerebral cortex because of the reduction in the calcium ATPase level and increase in levels of L-type Ca^2+^ channels expression (Cacna1d). The decrease in the levels of sodium/potassium, calcium, and magnesium ATPases could be due to the enhanced lipid peroxidation by free radicals in PCBs-treated rats.

 Oxidative stress and formation of free radicals are the major factors of the cytopathology of many neurogenerative disorders, where neuron displays oxidation and upregulation of oxidative defenses. PCB alters the activities of different antioxidant enzymes, membrane bound ATPases, and brain-specific CK activities and induces oxidative stress in hippocampus of male rats [31, 44].

 Quercetin has been reported to be effective in prevention of oxidative damage to DNA or to cell membrane [54]. One of the mechanism is that quercetin may stabilize lipid membranes and protect lipid peroxidation by free radicals scavenging mechanism thereby protecting tissues as evidenced in our earlier studies on rat seminal vesicles and ventral prostate [39, 40]. ROS scavenging activity of quercetin in the hippocampus of PCBs-exposed rats might be due to superoxide anion or hydroxyl radical alteration. Quercetin is a strong oxygen radical scavenger and also a good metal chelator. Johnson and Loo [55] showed that quercetin has a potent inhibitory activity against production of nitric oxide and TNF in lipopolysaccharide-stimulated Kupffer cells and also quercetin was shown to scavenge superoxide reperfusion injury. The antioxidant efficacy of quercetin may be due to its higher diffusion into the membranes [56] allowing it to scavenge oxyradicals at several sites through the lipid bilayer. It can be due to its pentahydroxyflavone structure allowing it to chelate metal ions *via* the orthodihydroxy phenolic structure, thereby scavenging lipid alkoxyl and peroxyl radicals [57].

It was also suggested that quercetin acts as an antioxidant by inhibiting oxidative enzymes such as xanthine oxidase, lipoxygenase, and NADPH oxidase. Inhibition of these enzymes is also responsible for the attenuation of oxidative stress as they play key roles in the initial process of free radical-induced cellular damage [58]. Further, it has been reported that quercetin metabolites can also inhibit peroxynitrite-mediated oxidation, similar to free quercetin [59]. Besides direct hydrogen-donating properties, more attention has been focused on the influence of quercetin on signaling pathways and its indirect interaction with the endogenous antioxidant defense system [60]. Quercetin, being the most potent of the tested flavonoids, has been found to increase the expression of C-glutamylcysteine synthetase with a concomitant increase in the intracellular glutathione concentrations [61]. Recently, Moskaug et al. [62] show that quercetin protects paracetamol induced oxidative stress and improves antioxidant enzymes activity in rat brain, plasma, lung, liver, kidney, heart, and testes. In the present study also, the same trend was observed in hippocampus of simultaneous quercetin treatment in PCBs-exposed animals. Quercetin can flux into brain regions, able to penetrate the blood brain barrier, and can protect the H_2_O_2_-induced neurotoxicity [63].

In conclusion, the decrements in cognitive functions were observed in several epidemiological studies of PCB intoxication but the relationship between PCB and impairment of cognitive functions is unclear. The inference of present study states that the hippocampal disruption of PCBs exposed rats, due to the significant increase of oxidative stress that results in the induction of hippocampal LPO, causes DNA fragmentation and decreases the activities of creatine kinase, acetyl cholinesterase, suggesting that ROS may be involved in the toxic effects of PCBs causing impaired learning and memory. However, supplementation of quercetin restored the biochemical and morphological alterations in hippocampus induced by PCBs. Thus this study enlightens the importance of quercetin in PCB-induced neurotoxicity and hippocampal dysfunction.

## Abbreviations

PCB: Polychlorinated biphenyls
H_2_O_2_: Hydrogen Peroxide
LPO: Lipid peroxidation
AchE: Acetyl cholinesterase
ATPase: Adenosine triphosphatases
Pcc: protein carbonyl content
CK: creatine kinase.
